# Biodegradation of the artificial sweetener acesulfame in biological wastewater treatment and sandfilters

**DOI:** 10.1016/j.watres.2016.11.041

**Published:** 2017-03-01

**Authors:** Sandro Castronovo, Arne Wick, Marco Scheurer, Karsten Nödler, Manoj Schulz, Thomas A. Ternes

**Affiliations:** a Federal Institute of Hydrology, Am Mainzer Tor 1, 56068 Koblenz, Germany; b DVGW Water Technology Center Karlsruhe (TZW), Department of Analyses and Water Quality, Karlsruher Str. 84, D-76139 Karlsruhe, Germany

**Keywords:** Acesulfame, Activated sludge, Sand filtration, Degradation, Transformation product, Wastewater tracer

## Abstract

A considerable removal of the artificial sweetener acesulfame (ACE) was observed during activated sludge processes at 13 wastewater treatment plants (WWTPs) as well as in a full-scale sand filter of a water works. A long-term sampling campaign over a period of almost two years revealed that ACE removal in WWTPs can be highly variable over time. Nitrifying/denitrifying sequencing batch reactors (SBR) as well as aerobic batch experiments with activated sludge and filter sand from a water works confirmed that both activated sludge as well as filter sand can efficiently remove ACE and that the removal can be attributed to biologically mediated degradation processes. The lab results strongly indicated that varying ACE removal in WWTPs is not associated with nitrification processes. Neither an enhancement of the nitrification rate nor the availability of ammonium or the inhibition of ammonium monooxygenase by *N*-allylthiourea (ATU) affected the degradation. Moreover, ACE was found to be also degradable by activated sludge under denitrifying conditions, while being persistent in the absence of both dissolved oxygen and nitrate. Using ion chromatography coupled with high resolution mass spectrometry, sulfamic acid (SA) was identified as the predominant transformation product (TP). Quantitative analysis of ACE and SA revealed a closed mass balance during the entire test period and confirmed that ACE was quantitatively transformed to SA. Measurements of dissolved organic carbon (DOC) revealed an almost complete removal of the carbon originating from ACE, thereby further confirming that SA is the only relevant final TP in the assumed degradation pathway of ACE. A first analysis of SA in three municipal WWTP revealed similar concentrations in influents and effluents with maximum concentrations of up to 2.3 mg/L. The high concentrations of SA in wastewater are in accordance with the extensive use of SA in acid cleaners, while the degradation of ACE in WWTPs adds only a very small portion of the total load of SA discharged into surface waters. No removal of SA was observed by the biological treatment applied at these WWTPs. Moreover, SA was also stable in the aerobic batch experiments conducted with the filter sand from a water works. Hence, SA might be a more appropriate wastewater tracer than ACE due to its chemical and microbiological persistence, the negligible sorbing affinity (high negative charge density) and its elevated concentrations in WWTP effluents.

## Highlights

• Acesulfame was biodegraded during wastewater and water treatment.
• Biodegradation of acesulfame occurred under oxic and denitrifying conditions.
• No relationship between the degradation rate and nitrifying activity was found.
• Sulfamic acid was identified as the main biotransformation product of acesulfame.
• Sulfamic acid is proposed as a new conservative wastewater tracer.

## Graphical abstract

**Figure:**
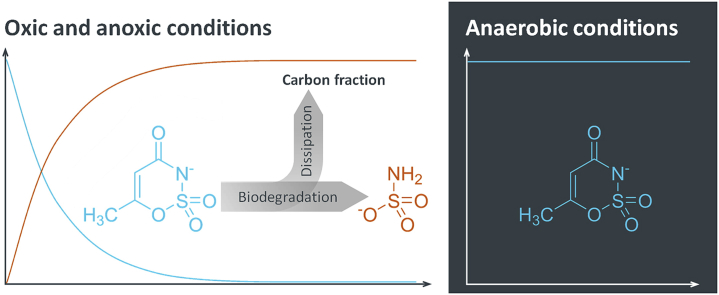

## 1 Introduction

The potassium salt of acesulfame (ACE) is widely used as an artificial sweetener in food, personal care products and pharmaceutical preparations. Latest estimates for the worldwide consumption of high-intensity sweeteners are about 117,000 metric tons with ACE ranked 5 in terms of sucrose equivalence (USDA, 2012). Due to advantageous synergistic properties with other sweeteners, its heat resistance and its long shelf-life, ACE is meanwhile approved in more than 100 countries (von Rymon Lipinski and Hanger, 2001). After ingestion and absorption from the gut, ACE is largely excreted unchanged in urine without undergoing any metabolism (Renwick, 1986, von Rymon Lipinski and Hanger, 2001) and is therefore present in wastewater in notable concentrations. On a worldwide scale, concentrations in the range of 10–100 μg/L in wastewater treatment plant (WWTP) influents and effluents have been observed in more than 20 countries (Arbeláez et al., 2015, Buerge et al., 2009, Gan et al., 2013, Kokotou and Thomaidis, 2013, Lee et al., 2015, Ordonez et al., 2012, Scheurer et al., 2009, Tran et al., 2014b, Xu et al., 2016) with a maximum concentration of 2.5 mg/L (Loos et al., 2013). As a consequence, ACE can be ubiquitously detected in anthropogenically influenced rivers and groundwater with concentrations up to the double-digit μg/L-range in surface waters, when these are highly impacted by wastewater discharge (Voloshenko-Rossin et al., 2015, Prasse et al., 2011, Engelhardt et al., 2013). ACE was even found in finished drinking waters in Switzerland, Germany, China and Canada with maximum concentrations of 2.6 μg/L, about 7 μg/L, 0.8 μg/L and 1.6 μg/L, respectively (Buerge et al., 2009, Prasse et al., 2011, Gan et al., 2013, Spoelstra et al., 2013).

It was reported that ACE can be degraded by technical processes applied in drinking water utilities such as photolysis by UV light (Gan et al., 2014, Sang et al., 2014, Scheurer et al., 2014) and ozonation (Scheurer et al., 2010), but the compound has been considered to be persistent to biodegradation in wastewater treatment plants (WWTPs) with activated sludge process as well as during soil passage (Buerge et al., 2009, Lange et al., 2012). Due to the assumed high persistence towards biodegradation and its high polarity ACE was also discussed as an ideal conservative tracer compound for domestic wastewater in surface water and groundwater (Buerge et al., 2009, Jekel et al., 2015). However, recent studies indicate that also biologically mediated reactions can occur and ACE can be degraded in microbially active compartments. Falås et al. (2016) observed a significant removal of ACE of up to 80% in bench-scale activated sludge sequencing batch reactors (SBR) at a hydraulic retention time (HRT) of 12 h and a solids retention time (SRT) of 10 d. The removal of ACE in these SBRs was attributed to biological degradation processes, since removal by sorption is negligible (Tran et al., 2015) and photodegradation in the opaque reactors could be excluded.

At certain German bank filtration sites Storck et al. (2015) observed a considerable decrease of ACE concentration. A comparison with conservative tracers (e.g. chloride) and the results from column experiments suggested that biological degradation rather than dilution and sorption was responsible for the concentration decrease. In another study Storck et al. (2016) investigated the persistence of ACE in laboratory experiments. While no significant removal was observed in flow-through column experiments, ACE was rapidly removed in a fixed-bed reactor under certain conditions.

The recent results from literature might indicate that biological degradation of ACE can only occur under specific environmental conditions. Especially redox conditions (Joss et al., 2004, Schmidt et al., 2007, Redeker et al., 2014), nitrifying activity (Fernandez-Fontaina et al., 2012) and C- and N-limiting conditions (Li et al., 2014) have been recently discussed to influence the biodegradation of those micropollutants for which a broad range of removal rates have been reported (e.g. trimethoprim and diclofenac). Tran et al. (2014a) demonstrated that the degradation of ACE by an enriched nitrifying culture can be enhanced by an increase of the initial ammonium concentration, which might indicate that autotrophic ammonia oxidizers play an important role in the biodegradation of ACE. However, further experiments of Tran et al. (2015) revealed a considerably slower removal of ACE by an enriched nitrifying culture compared to conventional activated sludge. Hence, a further systematic elucidation of factors, which trigger the biologically induced ACE degradation, is still needed.

Moreover, transformation products (TPs) of ACE formed by biodegradation are currently unknown. Gan et al. (2014) tentatively identified ten TPs of ACE, after incubating the compound, dissolved in deionized water, under simulated sunlight irradiation. The authors concluded that an enhanced degradation observed in unsterilized surface water samples might be explained by a joint effect of photo- and biodegradation. However, it remained unclear whether any of the identified photodegradation products in deionized water can also be formed by biologically mediated processes under environmental conditions.

The first aim of the current study was to scrutinize the persistence of ACE during activated sludge treatment of wastewater and sand filtration of surface water by examining its removal in a large number of nitrifying/denitrifying WWTPs and a full-scale sand filter used in a water works for the pre-treatment of surface water, respectively. Secondly, potential factors (redox conditions, nitrification activity) influencing ACE degradation were investigated by degradation experiments with lab-scale batches and bench-scale sequencing batch reactors. Thirdly, experiments conducted with activated sludge as well as discarded filter sand from waterworks were used to identify the TPs by means of high resolution mass spectrometry and to propose the major biologically mediated degradation pathway for ACE.

## 2 Materials and methods

### 2.1 Chemicals

Ammonium acetate, diclofenac sodium and ACE potassium (≥99% purity) were purchased from Sigma Aldrich (Schnelldorf, Germany). ACE-d_4_ potassium salt (≥95% purity), used as surrogate standard, was purchased from Toronto Research Chemicals (Toronto, Canada). Sulfamic acid (SA; ≥ 99% purity) was purchased from Carl Roth and Alfa Aesar GmbH (both Karlsruhe, Germany), *N*-allylthiourea (ATU; 98% purity) from Alfa Aesar (Ward Hill, USA). (NH_4_)_2_SO_4_ (≥99.5% purity), KH_2_PO_4_ (≥99.5% purity), NaOH (≥99% purity) and NaNO_3_ (≥99% purity) were purchased from Merck KGaA (Darmstadt, Germany). Acetoacetamide-*N*-sulfonic acid (ANSA) was kindly provided by Celanese (Frankfurt am Main, Germany). Methanol was supplied by Merck (Darmstadt, Germany) and ultrapure water was provided by an Arium 611 laboratory water purification system (Sartorius AG, Göttingen, Germany).

### 2.2 Sampling of municipal WWTPs and a full-scale sand filter

Sampling campaigns were conducted at thirteen municipal WWTPs (denoted by capital letters from A–M) located in Germany and Switzerland. All sampled WWTPs, except for WWTP H and M, are conventional plants using activated sludge treatment for nitrification and denitrification (SRT 10–35 d) and have a capacity between 25,500 and 440,000 population equivalents (PE). At WWTP H, secondary treatment is performed in two parallel-operated treatment units: a conventional activated sludge system and a fixed-bed reactor which receive approx. 50% of the primary effluent each. Both systems are designed to provide wastewater treatment by nitrification and denitrification. WWTP M is equipped with a biological treatment step which is cascaded into three compartments. The first denitrifying compartment is followed by an aerated step for COD removal. In both compartments the biomass consists of suspended sludge flocs. The third compartment is the nitrifying stage and contains carriers (35% filling ratio, 420 m^2^/m^3^) for biofilm growth. Four WWTPs (B, G, H and J) are equipped with sand filtration as a final treatment step. At each of the WWTPs six 24 h composite samples were taken from both influent (raw wastewater) and final effluent before discharge into the receiving water (i.e. after secondary clarification or sand filtration) within a period of 14 d. A summary about the individual WWTPs (e.g. capacity, SRT, biological treatment steps, COD removal, etc.) can be found in the supplementary information (SI, Table S1).

In order to assess the variability of ACE removal over time, one additional German WWTP in Eriskirch (ER) was sampled over a time course of two years. The WWTP ER has a capacity of 50,000 PE and biological treatment consists of conventional activated sludge treatment for denitrification and nitrification (SRT 12–18 d). The samples from the WWTP ER were taken as 24 h composite samples at dry weather conditions, in the middle of the week (Tuesday or Wednesday).

In addition to the WWTPs, a full-scale sand filter, used for slow sand filtration for the pre-treatment of surface water prior to artificial groundwater recharge, was sampled seven times over a period of three weeks. The study site was included to assess if ACE removal is limited to activated sludge processes in WWTPs or if remaining traces of ACE can be removed in other biologically active compartments like sand filters, e. g. when surface water is used for drinking water production. The respective drinking water treatment plant is described in detail in Nödler et al. (2013). Briefly, water from the River Ruhr is abstracted and subsequently subjected to basins for slow sand filtration (sand depth 1.5 m). The water is reclaimed after a short underground passage (2–4 d) and treated by applying ozonation, multi-layer filtration, and UV disinfection before it is supplied as drinking water. Samples taken for this study were the surface water and the reclaimed water after sand filtration and underground passage.

### 2.3 Bench-scale sequencing batch reactors (SBR)

A series of fully automated reactors with a volume of 12 L each were installed at the municipal WWTP L (SI, Table S1) located in Koblenz (Germany). This WWTP has an urban catchment of 220,000 PE and an average load of 220 mg/L BOD, 70 mg/L N_tot_ and 16 mg/L P_tot_. The bench-scale reactors were run in sequencing batch mode and fed with effluent of the primary clarifier of WWTP L (after screening, grit removal and sedimentation). Each reactor was equipped with sensors for the online measurement of fill level, pH, temperature, and concentrations of oxygen and ammonium. The process control was realized with a programmable logic controller (WAGO 750-880) and a SCADA system (Citect V7.2, Schneider Electric). Further reactor details are described in Falås et al. (2016).

To examine the degradation potential of ammonium-oxidizing bacteria two treatment chains were set up, each consisting of two reactors. In both chains the first reactors (R1 and R2) were operated as conventional activated sludge processes with nitrification (2/3 HRT) and denitrification (1/3 HRT) phases. Each of them was followed up by an aerobic post-treatment (R3 and R4, respectively). All reactors were running with a HRT of 12 h and a SRT of 12 d. Since biomass growth in the post-treatments was expected to be low as a result of the low nutrition load due to the pre-treatment, they were equipped with carriers to enable biofilm growth as compensation. R3 was additionally supplemented with an automated ammonium dosage to enrich ammonium-oxidizing bacteria and increase nitrification. The pH was maintained between 6.9 and 7.1 by an automated dosage of a NaOH-solution. After an initial start-up phase of four months, these reactors were monitored over a period of nine months, during which fourteen 72 h composite samples were taken. Furthermore, the sequencing batch-mode was paused for two days, during which the reactors were spiked with 5 μg/L ACE each and run in aerated batch mode to obtain degradation curves.

### 2.4 Laboratory batch experiments

Several laboratory-scale batch experiments were conducted to further elucidate the primary degradation of ACE and the formation of TPs in contact with activated sludge as well as filter sand. A general overview about the different setups of these batch experiments is provided in Table 1. All batch experiments with activated sludge were performed in triplicates in 500 mL amber glass bottles, which were continuously mixed by a magnetic stirrer. Temperature was maintained at 25 ± 2 °C. Matrices for batch experiments were collected at the municipal WWTP in Koblenz (WWTP L). Influent was taken after the primary clarifier, effluent from the discharge of the WWTP. Activated sludge was taken from the nitrification basin (HRT 6 h, SRT 12 d) or from the aerobic bench-scale reactor R1 described above. The matrices were immediately transported to the laboratory and experiments were started at the same day.

The first test approach (A) was designed to distinguish biotic from abiotic degradation processes and to elucidate the influence of the initial ammonium concentration as well as the ammonia monooxygenase enzyme (AMO) activity. Therefore, five different treatments were set up, all inoculated with activated sludge from the WWTP L. The sludge was diluted either with WWTP influent or effluent and spiked up to a total concentration of 50 μg/L ACE, which corresponds to concentration ranges commonly found in municipal wastewater. One setup was autoclaved and used as sterile control to check for abiotic degradation processes. Another setup was supplemented with 20 mg/L NH_4_-N and a third setup was supplemented with 5 mg/L ATU as inhibitor of the AMO, to suppress the nitrification process. All lab-scale experiments were constantly aerated.

The aim of the second approach (B) was to examine the degradation potential under different redox-conditions. Therefore, activated sludge from the WWTP L was incubated under oxic conditions with constant aeration, anoxic/denitrifying conditions without aeration and addition of 200 mg/L NO_3_-N as substituting electron acceptor and anaerobic conditions without aeration.

The third approach (C) was performed to identify transformation products (TPs) and to propose the major degradation pathway of ACE. Therefore, batches were incubated with activated sludge from SBR under constant aeration, spiked with 40 mg/L ACE and compared to a control setup without spike. The spiked batches were re-spiked with 40 mg/L ACE after 14 d and 21 d.

In a fourth approach (D) activated sludge from SBR was repeatedly washed and re-suspended in 50 mM phosphate buffer (KH_2_PO_4_) to remove the natural wastewater background. The lab-scale experiments were spiked with either 400 μg/L ACE or 400 μg/L ANSA and incubated under constant aeration.

Experiments with activated sludge were supplemented by batch tests with filter sand (E). The sand was taken from a pilot plant sand column used for simulating slow sand filtration as a pre-treatment of surface water prior to drinking water production. The column was filled with technical sand of the River Rhine to a height of about 80 cm (column diameter 45 cm). As inoculum the upper layer was mixed with native sediment from the River Ruhr (10% by mass). The column was loaded with water from the River Ruhr, which was pre-filtered over gravel. This sand column has been previously described to be capable of removing ACE. Details about the column setup and its efficiency for ACE removal are described by Drechsel et al. (2015). An aliquot of this sand was stored at 4 °C (together with overlaying water from the experiments) until its application within the experiments presented here: About 130 g of wet sand was weighted in 1 L clear glass bottles, which were filled with 800 mL deionized water and 200 mL potable water. The experiments were aerated to ensure oxic conditions and run in duplicates. Over the course of the study several experiments (E1–E2) were conducted with different concentration levels (10 μg/L to 10 mg/L) of ACE. Unspiked control batches were run in parallel to check for background contaminations of SA and for water chemical parameters.

### 2.5 Preparation of samples and spike solutions

All samples were filtrated (0.45 μm, Whatman SPARTAN Syringe Filter, regenerated cellulose, GE Healthcare Life Sciences) immediately, spiked with the internal standard ACE-d_4_ and either kept refrigerated (4 °C) and analysed within seven days or stored frozen (−20 °C) until analysis. All spike solutions were prepared as aqueous stock solutions to avoid any impact of organic solvents (potential carbon source, effects on microbial community).

### 2.6 Quantitative analysis and identification of TPs

Quantitative analysis of samples was conducted by either liquid chromatography (LC) or ion chromatography (IC) coupled to tandem mass spectrometry (MS/MS). High-resolution mass spectrometry and MS^n^ fragmentation experiments were performed for the detection, identification and characterization of ACE TPs.

In addition to mass spectrometry other detection techniques were applied for the basic parameters dissolved organic carbon (DOC), NO_3_-N and NH_4_-N. All methods are described in detail in the supporting information (SI, S1).

### 2.7 Calculation of degradation rate constants

Except for the high spike experiments (c_0_ ≥ 10 mg/L), the degradation of ACE in batch experiments conducted with activated sludge was modelled according to Schwarzenbach et al. (2005) by pseudo-first-order kinetics:

$$\frac{dc_{ACE}}{dt}=-k_{biol}\cdot X_{ss}\cdot c_{ACE}$$

where $X_{ss}$ is the sludge concentration [g_SS_/L], c_ACE_ the ACE concentration [μg/L] and $k_{biol}$ the pseudo-first-order rate constant [L/(g_SS_·d)].

## 3 Results and discussion

### 3.1 Removal of ACE in WWTPs

**Fig. 1:**
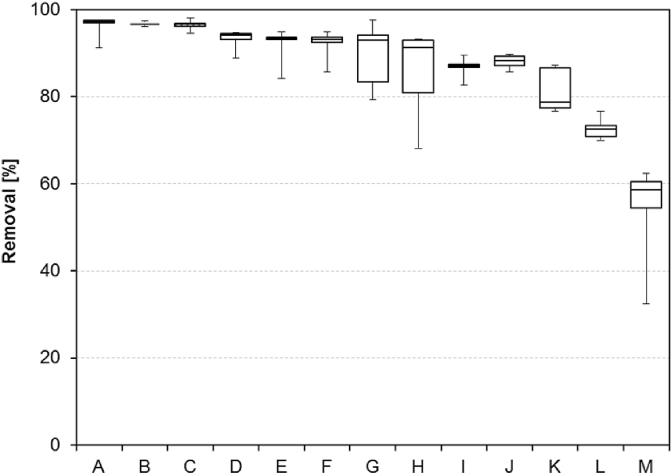
ACE removal [%] in thirteen WWTPs (A-M) presented as box-and-whisker plots (median, 25% quantile, 75% quantile, maximum and minimum). WWTPs were sorted from highest to lowest removal efficiency.

The sampling campaign at 13 WWTPs revealed influent concentrations ranging from approx. 20 μg/L (WWTP G) to 80 μg/L (WWTP K) with a median of 38 μg/L. This is in good accordance to literature data reporting a wide range of influent concentrations between only a few μg/L (Ryu et al., 2014, Subedi and Kannan, 2014) and more than 100 μg/L (Arbeláez et al., 2015, Nödler et al., 2013). However, in contrast to most previous reports ACE concentrations were considerably reduced by conventional biological wastewater treatment in all sampled WWTPs (Fig. 1 and SI, Table S1). The lowest median removal of ACE was observed in WWTP M, which uses a hybrid biofilm-activated sludge process for denitrification and nitrification, and was still as high as 59%. A maximum median removal of 97% was achieved in three WWTPs (A, B and C) using conventional activated sludge treatment with denitrification and nitrification. The results strongly indicate that a considerable removal (most likely degradation) of ACE in nitrifying/denitrifying WWTPs is not as rare as previously expected. So far, no relationship between ACE removal and WWTP characteristics (SI, Table S1) was observed.

Since previous studies showed that removal of ACE by sorption is negligible (Storck et al., 2016, Tran et al., 2015), the observed removal in WWTPs can be most likely attributed to degradation processes rather than sorption to activated sludge. This was further confirmed by the closed mass balance obtained in the batch experiments conducted within this study (see Section 3.5).

**Fig. 2:**
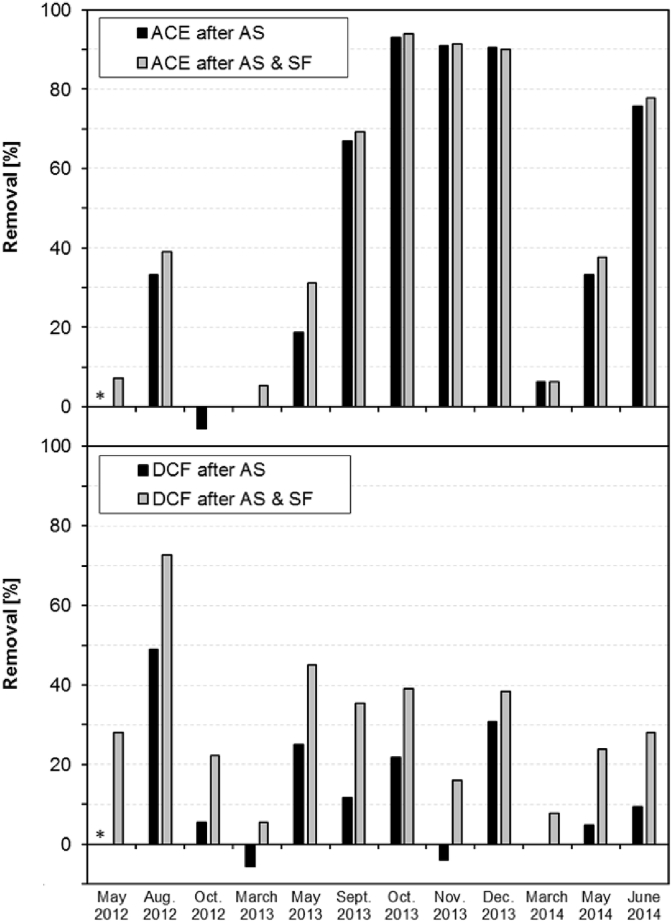
Removal [%] of acesulfame (ACE) and diclofenac (DCF) in the WWTP ER after activated sludge treatment (AS) and subsequent sand filtration (SF) at twelve sampling events over a time course of two years (*: no data).

To address also the possible temporal variation of ACE removal, twelve 24 h composite samples were taken at the WWTP ER over a period of two years between May 2012 and June 2014. Biological treatment at this WWTP (capacity 50,000 PE, 17,300 m³/d) consists of an activated sludge treatment (SRT 12–18 d) followed by a secondary clarifier, flocculation and a sand filtration step. The overall median removal of ACE in this WWTP was only 39% and thus substantially lower than examined during the WWTP sampling survey described above. The main removal already occurred during activated sludge treatment (median of 33%), while the flocculation and sand filtration only contributed about 6% to the overall removal. During the sampling period the removal efficiencies varied strongly between <20% and a maximum removal of more than 90% (Fig. 2, top). The results indicate that the capability of activated sludge for ACE degradation can underlie strong variations. The variations in the removal of ACE are surprising, but are in accordance with only recently published results of the State Agency for Environment, Measurements and Nature Conservation of the federal state of Baden-Württemberg (Germany). For the WWTP in Heilbronn (Southwestern Germany) an ACE elimination between 6 and 88% was reported (LUBW, 2014). The authors observed no correlation with temperature or season. Like in the WWTP ER, sampling events with minimal ACE elimination were followed by ones with >80% ACE removal, suggesting that removal efficiencies are not permanently established, but are snap-shots of short-term boundary conditions.

In order to examine whether a good removal of ACE was accompanied with generally improved micropollutant degradation, ACE removals were also compared with those of diclofenac (Fig. 2, bottom). Diclofenac was chosen, since for WWTPs a broad range of removal efficiencies has been determined in a previous publication (Vieno and Sillanpää, 2014). However, diclofenac and ACE removal did not correlate at all and it even occurred that at sampling events with the highest ACE removal, no diclofenac removal during activated sludge treatment was observed. In contrast to ACE, subsequent sand filtration considerably contributed to further diclofenac removal in this WWTP. Hence, ACE removal might be connected to the presence and activity of rather specific microbial species and enzymes underlying distinct dynamics in the activated sludge microbial community. Further sampling campaigns with a higher temporal resolution along with the characterization of the microbial community and/or genes, transcripts or enzymes by meta-omics methods are needed to gain a deeper understanding of the highly variable ACE elimination in some WWTPs or the differences between environmental compartments.

### 3.2 ACE removal in slow sand filtration

In order to assess the potential of microbial communities present in other environmental compartments to degrade ACE and to confirm the results obtained in the pilot-plant slow sand filtration by Drechsel et al. (2015), a full-scale slow sand filter was included as an additional study site. Like the pilot plant, the sand filter receives surface water from the River Ruhr. During the sampling campaign ACE concentrations in the river water ranged between 0.11 μg/L and 0.57 μg/L (mean 0.37 μg/L), while in the reclaimed water after sand filtration and underground passage (total residence time 2–4 d) the compound was measured between 0.08 μg/L and 0.13 μg/L (mean 0.11 μg/L, SI, Fig. S1). Comparing the mean values, ACE concentrations were decreased by about 70%. The narrower concentration range in the reclaimed water displays the ability of soil passages/bank filtration sites to buffer concentration peaks (and lows). Although it is difficult to distinguish between degradation and dilution with land borne groundwater, there seems to be evidence that ACE was biologically degraded also at this site. It has to be noted, that the sampling campaign coincided with an extremely high discharge of the Ruhr River of up to 370 m^3^/s at one sampling day, so considerable dilution of the ACE concentrations were prevailing at this time. A recent study (AWWR/Ruhrverband, 2014) confirmed that ACE concentrations in the River Ruhr can reach several μg/L. However, also at these times with ACE values in the μg/L-range in the surface water, concentrations in the collection well were measured in the same range as observed during our sampling campaign by the local water utility. This suggests that our observations most likely even underestimate ACE removal efficiency at this site.

### 3.3 Elucidation of potential factors influencing ACE degradation

#### 3.3.1 Primary degradation of ACE and impact of nitrifying activity

**Fig. 3:**
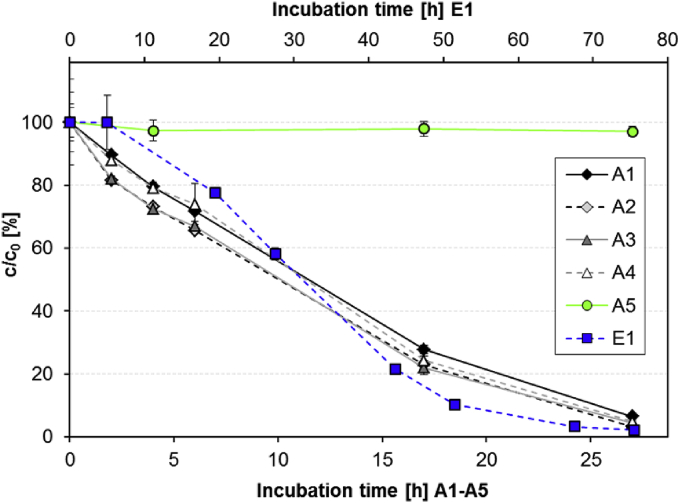
ACE removal in **activated sludge** (triplicates per treatment, error bars represent the standard deviation: **A1** mixed 1:1 with WWTP-influent; **A2** mixed 1:1 with WWTP-influent and ATU-addition; **A3** mixed 1:1 with WWTP-effluent; **A4** mixed 1:1 with WWTP-effluent and NH_4_^+^ addition **A5** mixed 1:1 with WWTP-influent, sterile) and **sand** (duplicates, error bars represent minimum and maximum values: **E1** with deionized water + potable water (80/20, v/v)).

Several batch experiments with aerated activated sludge (c_0_ = 50 μg/L ACE) and filter sand were conducted in parallel to confirm that the removal observed in WWTPs and the full-scale sand filter is caused by degradation processes. Furthermore, the influence of nitrifying activity on ACE removal was investigated. In all biologically active treatment setups with activated sludge (A1–A4) ACE was removed by more than 94% within 27 h (Fig. 3), affirming the ACE removal observed in the WWTPs. Since ACE was persistent in the autoclaved control (A5), the removal of ACE in contact with activated sludge could be attributed to microbial degradation processes. Degradation could be well described by pseudo-first-order kinetics and similar rate constants ranging from 1.27 ± 0.07 to 1.57 ± 0.18 L/(g_SS_ d) were determined for all the different treatments.

In lab-scale experiments with filter sand (setup E1, c_0_ = 10 μg/L) ACE was also efficiently degraded (Fig. 3). Even though the biomass can be assumed to be significantly lower, the 50% dissipation time (DT_50_ ∼ 30 h) was only about three times higher than in the experiments with activated sludge (DT_50_ ∼ 12 h). After a lag-phase of about 20 h the degradation could be well described by first-order kinetics with a rate constant of 1.62 1/d.

The batch experiments A1 and A4 contained an initial NH_4_-N concentration of 15.3 and 19.4 mg/L, respectively. Complete removal of NH_4_-N within the first 6 h of incubation (SI, Table S3) confirmed an extensive nitrification in these batches. However, the degradation rate in A1 (1.27 ± 0.07 L/(g_SS_ d)) and in A4 (1.42 ± 0.17 L/(g_SS_ L)) were not significantly different from the degradation rate in batch A3 (1.45 ± 0.20 L/(g_SS_ L)), which contained less than 0.08 mg/L NH_4_-N at the beginning of the experiment. Moreover, also the successful suppression of nitrification via the inhibition of AMO by ATU addition to batch A2 (initial NH_4_-N concentration of 14.9 mg/L, no removal throughout the experiment, see SI, Table S3), had no significant influence on ACE removal. This was a first indication that nitrification processes in general and the activity of the AMO-enzyme in particular are not decisive for ACE degradation.

To assess whether ACE removal can be improved by increasing the nitrification rate, a bench-scale SBR (R3) with carriers (40% filling ratio) and continuous ammonium dosage (5–20 mg/L) was established to achieve conditions favorable for autotrophic ammonium nitrifiers. This reactor was installed as an aerobic post-treatment of a conventional activated sludge treatment with denitrification and nitrification (R1) to reduce the load of organic carbon, thereby providing a competitive advantage towards heterotrophic organisms. For comparison, a parallel reactor series was operated with identical pre-treatment (R2), but without ammonium dosage to the post-treatment (R4). A measurement of the potential nitrification capability under constant ammonium dosage confirmed an increased NO_3_^−^ formation rate in the supplemented post-treatment R3 (294 mg/(L d)), which was more than 25 times higher than in the post-treatment R4 operated without ammonium addition (11 mg/(L d)) and even slightly higher than in the pre-treatment R1 (214 mg/(L d)).

**Fig. 4:**
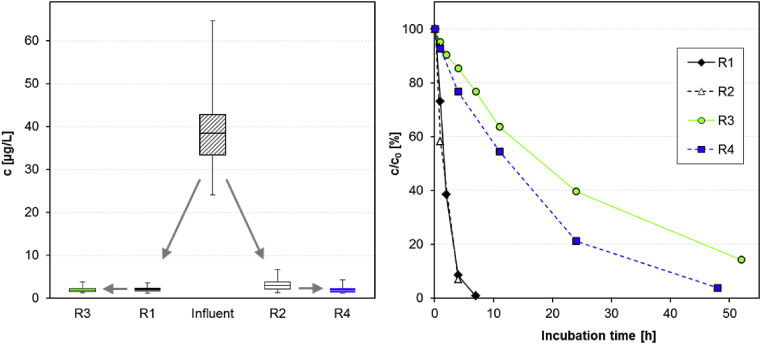
ACE removal in bench-scale reactors. **Left**: running in sequencing batch mode as conventional activated sludge process (R1, R2) and subsequent aerobic post-treatments with (R3) or without (R4) ammonium dosage, monitored over a period of 9 month with 14 samplings (box-and-whisker plots: median, 25% quantile, 75% quantile, maximum and minimum). **Right**: same reactors running in batch mode with initial spike of 5 μg/L ACE.

The monitoring of ACE in sequencing batch mode (Fig. 4, left) revealed that no improved degradation was obtained by the increase of the nitrification rate. While in both pre-treatments ACE was removed clearly more extensive (R1: 95%; R2: 92%) than in the post-treatments, the comparison between both post-treatments showed even less removal in the ammonium supplemented R3 (<20%) than in the complementary reactor R4 (37%). These results were confirmed in a spiked batch experiment in the same reactors (Fig. 4, right). Also here the observed ACE removal in the post-treatments (DT_50_: 18.4 h in R3; 12.7 h in R4) was considerably slower than in the conventional treatment reactors (DT_50_: 1.7 h in R1; 1.5 h in R2) and the enhanced nitrification rate in R3 did not lead to an improved ACE removal in comparison to the post-treatment control (R4) operated without ammonium dosage.

In summary the experimental results did not give any indication that the ACE removal efficiency is associated with nitrification processes. Neither did enhanced nitrification activity promote ACE removal, nor did the availability of ammonium or the inhibition of AMO affect the degradation. Therefore, in contrast to the assumption that an increased biodegradation efficiency of ACE could be expected in wastewater treatment with high nitrification rates (Tran et al., 2014a), the results of the current study strongly indicate that nitrification rate by itself is not a crucial factor for ACE removal in biological wastewater treatment.

#### 3.3.2 Incubation under oxic, anoxic and anaerobic conditions

**Fig. 5:**
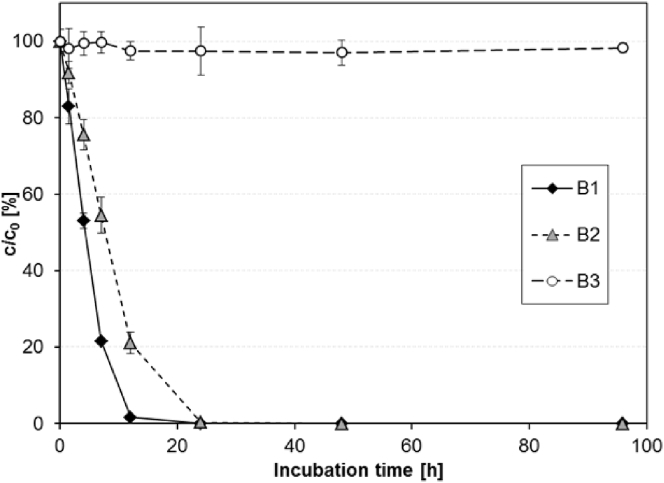
ACE removal under various redox-conditions in activated sludge from the WWTP L: incubated under oxic (B1), anoxic (B2) and anaerobic (B3) conditions, error bars represent the standard deviation (n = 3).

The influence of redox conditions on the ACE removal was investigated in laboratory batch experiments with activated sludge from the WWTP L. While one batch (B1) was incubated under oxic conditions with permanent aeration, a second batch (B2) was incubated under an argon atmosphere and 200 mg/L NO_3_-N were added to establish anoxic (denitrifying) conditions. Under both oxic and anoxic conditions ACE was rapidly removed (Fig. 5) at rates of 5.04 ± 0.12 and 3.53 ± 0.09 L/(g_SS_ L), respectively. These rates were even higher than those determined in the previous experiments A1–A4 (see 3.5.1) and further supported the finding that ACE removal in full-scale plants can underlie considerable changes over time.

In contrast, under strictly anaerobic conditions in a third batch (B3) incubated under an argon atmosphere without NO_3_^−^ addition, no ACE removal was observed over an incubation period of 96 h. These results are consistent with those from Falås et al. (2016) who observed considerable ACE removal of up to 80% in an aerobic SBR, but no removal in different anaerobic SBRs operated under methanogenic, sulfate-reducing and iron-reducing conditions for more than one year. Moreover, also in column experiments with undisturbed sediment cores from the infiltration zone of a German bank filtration site (Burke et al., 2014), removal of ACE was restricted to the upper layers where oxic and denitrifying conditions were prevailing. In contrast, the authors observed no removal in the subsequent manganese reducing zone.

It can be concluded that ACE can be degraded under both oxic and denitrifying conditions, but is rather persistent under strictly anaerobic conditions in the absence of both oxygen and nitrate. Hence, the primary reaction in the degradation pathway of ACE does not rely on the availability of free dissolved oxygen. Even though it cannot be fully excluded that degradation pathways under oxic and anoxic conditions are different, the results also indicate that for example (mono-)oxygenase reactions are most likely not involved in the primary degradation of ACE.

### 3.4 Identification of transformation products of ACE

**Fig. 6:**
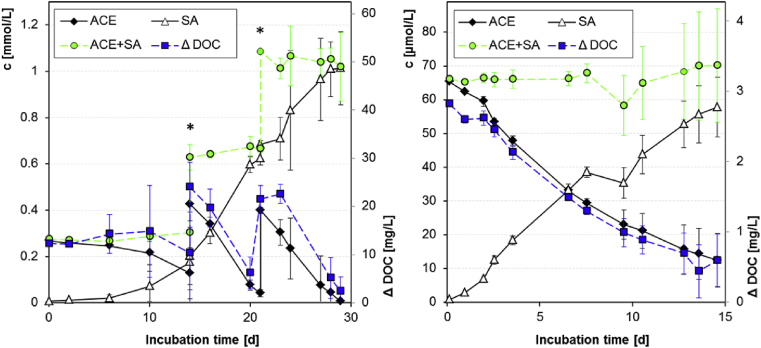
Mass balance of ACE and SA in comparison to the DOC difference between spiked and unspiked batches (Δ DOC). Primary and secondary y-axes are equivalently scaled, i.e.: c(ACE) ≙ nominal DOC(ACE). **Left**: Mean values of triplicates in activated sludge from SBR, error bars represent the standard deviation. ACE was spiked to an initial concentration of 40 mg/L. The asterisks mark a re-spike after 14 and 21 d. **Right**: Mean values of duplicates in filter sand (c_0_ = 10 mg/L), error bars represent the minimum and maximum values.

For the identification of transformation products (TPs) diluted activated sludge was spiked with an elevated concentration of 40 mg/L (≙ 250 μmol/L) ACE (treatment setup C1, Table 1) and re-spiked after 14 d and 21 d. Considerable degradation of ACE could be observed after a lag phase of approx. one week (Fig. 6). The degradation rate increased over time and followed approximately zero order kinetics with a rate of about 80 μmol/d after two weeks. The results suggested an adaption of the microbial sludge community and indicated a metabolic use of ACE at the mg/L concentrations.

Analysis with IC-LTQ-Orbitrap MS revealed the formation of five TPs: TP96, TP180a, TP180b, TP178 and TP192. A list of the retention times, precursors and product ions from MS and MS^2^ spectra can be found in the supplementary information (SI, Table S7). Two of the TPs (TP96 and TP180a) could be unambiguously identified as sulfamic acid (SA) and acetoacetamide-*N*-sulfonic acid (ANSA) by comparison of retention times and MS fragmentation spectra with authentic reference standards. SA has also been described in literature as a TP of ACE formed by photodegradation (Sang et al., 2014, Scheurer et al., 2014) and ozonation (Scheurer et al., 2012) and is used as a raw material for the synthesis of ACE (Haber et al., 2006). ANSA has also been tentatively identified as TP resulting from UV irradiation of ACE (Sang et al., 2014). Moreover, Scheurer et al. (2014) and Gan et al. (2014) also described TPs with m/z 180 and a MS^2^ spectrum comparable to that of ANSA as a product of photolysis. In addition to photolysis, it was found that ANSA can be formed in traces as decomposition product after storage of ACE under extreme conditions (temperature 100–120 °C, pH < 2.5, stored 2–6 months; described in Kreiling and Mayer, 1991). However, considering the experimental procedure a chemical formation of ANSA by UV-light or high temperatures and low pH is rather unlikely in the current study and suggests that ACE can also be transformed to SA and ANSA by a microbially catalyzed reaction.

**Fig. 7:**
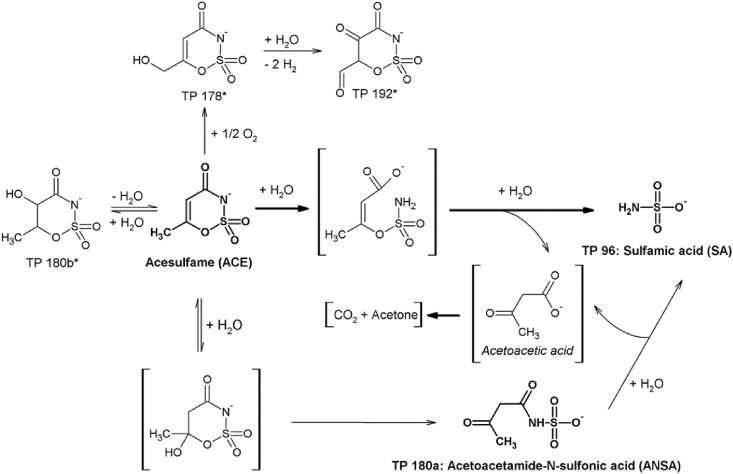
Proposed degradation pathway of ACE: TPs which were unambiguously identified by reference standards are printed in bold. Tentative structures of further TPs detected are marked with asterisks. Proposed intermediates which could not be detected are shown in parentheses.

For other TPs (TP180b, TP178 and TP192) chemical structures were proposed (Fig. 7) based on the accurate mass of the parent and product ions but could not be further confirmed, since reference standards were not available and quantities were too low for structural elucidation by NMR. However, the identified TP SA was by far the most dominant TP. All other TPs did not exceed 1% of the initial peak area of ACE. More details about the structural elucidation are given in the supplementary information (SI, S2).

### 3.5 Mass balance of ACE and SA

Quantitative analysis of ACE and SA on a molar basis revealed a closed mass balance and showed that ACE was transformed almost completely to SA (Fig. 6). Throughout the entire test period the sum of the molar concentrations of ACE and SA accounted for 98–118% of the spiked ACE concentrations (SI, Fig. S2). This confirmed that the quantities of the other detected TPs which still contained the SA moiety were negligible and that these TPs were either short-living intermediates in the transformation of ACE to SA or TPs of less relevant side reactions.

To further elucidate the fate of the carbon fraction of ACE, the DOC concentrations were measured in spiked batches and compared to those in unspiked controls. The difference of DOC concentrations between spiked and unspiked batches (Δ DOC) was determined as an indicator for DOC originating from ACE and its organic TPs. The initial addition of 40 mg/L ACE led to a DOC increase of 12.2 ± 0.3 mg/L, which corresponds to a recovery of 103 ± 2% and confirmed the feasibility of the approach. The Δ DOC considerably dissipated after each ACE spike and during the entire test period the dissipation trend was rather similar to that of the nominal DOC calculated from measured ACE concentrations (Fig. 6, left). Only a small offset was observed between the Δ DOC and the nominal DOC which could be mainly explained by a slight increase of the Δ DOC during the first 10 d of incubation due to a stronger increase of the natural background DOC in the spiked samples in comparison to the unspiked control samples. However, the amount of Δ DOC removed after the second spike between day 14 to day 20 (17.7 mg/L, 74%) and the third spike between day 21 to day 29 (19.1 mg/L, 89%) agreed very well with the corresponding amount of the nominal DOC of the measured ACE removed after the second (16.7 mg/L, 82%) and third spike (18.9 mg/L, 99%). These results confirmed that the major part of the organic fraction of ACE was mineralized, incorporated into the microbial biomass, volatilized or less likely sorbed to sludge. This was in accordance with the finding that no other TPs than the dominant inorganic TP SA could be detected at relevant amounts (>1% of the initial peak area of ACE) by the mass spectrometric approaches.

Comparable results were also achieved in lab-scale experiments with filter sand (setup E2, Table 1) at an initial ACE concentration of 10 mg/L (Fig. 6, right). About 80% of the initial concentration of ACE was removed at the end of the test duration of about 14.5 d. In accordance to the results from batch experiments with activated sludge described above, ACE removal in contact with the filter sand was also accompanied by the formation of SA. An almost closed mass balance between 88% and 106% throughout the whole incubation period supported an almost complete transformation of ACE to SA. Moreover, the removal of ACE was also accompanied by a simultaneous decrease of the DOC. At the end of the incubation period the Δ DOC in the ACE batches was reduced by about 79%, which was in very good accordance to the ACE decrease. This gave further evidence that independently of the environmental matrix the degradation of ACE leads to a quantitative formation of SA, while the organic fraction of ACE is immediately mineralized, incorporated into the biomass and/or volatilized.

### 3.6 Degradation pathway

The quantitative transformation of ACE to SA showed that the sulfonamide was stable and not hydrolyzed as for example predicted by the pathway prediction system EAWAG-BBD/PPS. In contrast, the formation of SA implies that both the sulfonic acid ester and the amide bond of ACE can be cleaved. Since the hydrolysis of the sulfonic ester would lead to the TP ANSA which was identified in the high spike batch experiments with activated sludge, ANSA was assumed to be a potential transient intermediate in the degradation pathway of ACE to SA (Fig. 7). Indeed, degradation batch experiments with ANSA conducted with washed activated sludge (D2, Table 1) showed that ANSA was also predominantly transformed to SA (SI, Fig. S3). However, the dissipation of ANSA and the formation of SA were even slower than the dissipation of ACE and the SA formation in parallel batch experiments spiked with ACE (D1, Table 1) (SI, Fig. S4). Hence, if ANSA was a relevant precursor of SA in the degradation pathway of ACE, it should have accumulated to a certain extent in degradation experiments with ACE. In contrast, only trace amounts of ANSA were observed in the high spike experiments where the initial concentration of ACE was 40 mg/L. These results strongly indicated that ANSA is only a minor intermediate of SA and that the relevant transformation of ACE to SA proceeds not via ANSA, but another so far unknown pathway. Since the mass balance of ACE and SA was closed throughout the incubation period (see 3.5) and the coupling of different chromatographic methods (reversed-phase and ion chromatography) with Orbitrap-MS did not lead to the detection of further potential intermediates, it can be assumed that this pathway involves rather short living intermediates.

One hypothesis is that the amide moiety is hydrolyzed first, which leads to the formation of an alkyl sulfamate bearing a carboxylic group (Fig. 7). Even though this compound also exhibits an exact mass of 179.9972, neither TP180a (identified as ANSA based on a comparison with a reference standard) nor TP180b (MS^2^ fragments of TP 180b strongly indicated that the amide bond was still present) could be allocated to this proposed structure. Hence, it has to be assumed that the alkyl sulfamate intermediate formed by the proposed initial amide cleavage is very short-living and rapidly hydrolyses to give the observed TP SA and acetoacetic acid. For aryl sulfamate anions chemical non-enzymatically catalyzed hydrolysis has been described by an S_N_1 mechanism with SO_2_NH as an intermediate which immediately reacts with water as a nucleophile to form SA (Spillane et al., 2011, Williams et al., 2014). However, also a very fast enzymatically catalyzed cleavage of the sulfamate ester is conceivable. For example, it has been described that S-OR bonds of sulfamates can be cleaved by sulfatases (EC 3.1.6.-) leading to the formation of SO_2_NH which can react with water to SA, but also with catalytic residues in the active site (Ahmed et al., 2002, Williams et al., 2014).

In contrast to SA, acetoacetic acid, the second product of the hypothesized hydrolysis of ACE could not be detected. This can be explained by its rather low stability due to chemical and enzymatic decarboxylation (Hay and Bond, 1967, Wolfenden et al., 2011) leading to the formation of acetone and CO_2_.

For a confirmation of this hypothesized degradation pathway additional highly time-resolved experiments, in particular with ^14^C-labeled ACE are required. These experiments might allow to detect the precursors of SA and to track the fate of the organic fraction of ACE. However, this was out of the scope of the current study and has to be addressed in follow-up studies.

### 3.7 Environmental relevance of ACE transformation to SA

#### 3.7.1 Transformation of ACE to SA at environmentally relevant concentrations

It has been shown for other biodegradable substances like trimethoprim or ibuprofen that the initial concentration of a micropollutant can affect degradation kinetics (Collado et al., 2012) or even completely change its degradation pathway (Jewell et al., 2016). Therefore the quantitative formation of SA had to be confirmed for environmental relevant concentrations. Due to high background concentrations of SA in wastewater (see 3.7.2), batch experiments with native activated sludge were not suitable for this purpose. In order to considerably lower the background concentration of SA, the activated sludge was repeatedly washed and resuspended in 50 mM phosphate buffer (KH_2_PO_4_). ACE was spiked at an initial concentration of 400 μg/L (∼2.5 μmol/L), which was a 100-fold lower than in the high spike experiments but still higher than in WWTP influents to exclude any interference with remaining SA concentrations. In contact with the washed sludge, ACE was also quite rapidly removed. After 72 h of incubation ACE was below the LOQ of 2 μg/L corresponding to a removal of more than 99% (SI, Fig. S4). Similar to the high spike experiments, the removal of ACE lead to a parallel formation of SA and the mass balance of ACE and SA was closed (82–99%) throughout the entire incubation period. No other TPs than SA could be detected. This was in accordance to the negligible amounts of the other TPs identified in the high spike experiment. Even though the initial ACE concentration was still higher than in influents of WWTPs, the results strongly indicate that the results from the high spike experiments can also be transferred to environmental relevant concentrations and that ACE removal in WWTPs also leads to the formation of SA.

#### 3.7.2 Occurrence of SA in WWTPs

The occurrence of SA in municipal wastewater was studied in 24 h composite samples taken from the influent and effluent of the WWTP L as well as two other nitrifying WWTPs located in Germany. While the WWTP L uses activated sludge treatment for denitrification and nitrification, biological treatment in the other two WWTPs consists of activated sludge treatment for CSB removal (SRT 3–4 d) followed by a trickling filter for nitrification. The influents of the three WWTPs contained rather high SA concentrations of 0.27 ± 0.06, 1.6 ± 0.1 and 2.0 ± 0.4 mg/L. This can be explained by the extensive use of SA in acid cleaners in a large variety of household and industrial applications (Motamedi et al., 2013). Since the influent concentrations of SA were up to 40 times higher than the influent concentration of ACE (∼0.05 mg/L), the amount of SA formed by the degradation of ACE could not be differentiated from the high background level of SA. The measured effluent concentrations of 0.27 ± 0.11, 1.4 ± 0.1 and 2.3 ± 0.4 mg/L were not significantly different from the influent concentrations. This was a first indication that SA is rather stable under aerobic conditions and that neither nitrifying activated sludge nor biofilms in trickling filters are capable of removing SA to a significant extent. These results were also in accordance with those from the degradation batch experiments with activated sludge and filter sand where SA was stable over a period of 29 and 14.5 d, respectively (see 3.5).

It can be concluded that municipal WWTPs are an important point source for the discharge of SA into surface waters. However, assuming that at least the order of magnitude of SA concentrations detected in the three WWTPs is representative for other municipal WWTPs, it implies that the degradation of ACE in WWTPs does not significantly contribute to the concentrations of SA in WWTP effluents.

Even though SA is a high production volume chemical with a broad range of applications, to the best of our knowledge this is the first time that the occurrence of SA in municipal wastewater has been reported. It has to be noted, that the widespread use of SA goes along with a comparatively low toxicity and so far toxicological studies indicate no risk of SA for human health. The US Food and Drug Administration (FDA, 1972) even approved SA as a GRAS (generally recognized as safe) substance for usage in food packaging. The Pesticide Action Network (PAN) database considers the compound only to be slightly acute toxic (Kegley et al., 2014) to aquatic organisms, based on two studies with fathead minnow (*Pimephales promelas*), one of them providing LC_50_ (96 h) values of up to 70.3 mg/L (Curtis and Ward, 1981). Regarding aquatic toxicity also the European Chemicals Agency (ECHA, 2016) lists several endpoints for different trophic levels; e. g. a no observed effect concentration (NOEC) of >60 mg/L is reported in an early life stage test with *Danio rerio*, in which no negative effects on hatching, mortality, body length and weight were observed. Furthermore, the assumption is made, that the LC_50_ observed by Curtis and Ward (1981) was primarily caused by the acidic character of the compound and the low pH in the test rather than by the anionic species predominantly encountered in the aquatic environment. Tests with aquatic invertebrates and algae resulted in similar concentration ranges for the endpoints tested. Furthermore, SA showed no inhibitory effect in a sludge respiration inhibition test and a NOEC of >200 mg/L (highest test concentration) was assessed (ECHA, 2016). Thus, even though concentrations in the mg/L range were detected in wastewater, by the current state of knowledge no negative effect on the aquatic environment is to be expected.

### 3.8 Consequences of the study for use of ACE as a conservative wastewater tracer

As demonstrated, ACE degradation in WWTPs and slow sand filtration is not a rare phenomenon and can be reproduced in laboratory experiments. Moreover, a study of Storck et al. (2015) gave evidence that at specific sites ACE can be also degraded during bank filtration. Even though at the present stage no clear forecast can be made regarding the probability of its biodegradation in a particular environment, these results strongly indicate that ACE is not a generally appropriate wastewater tracer for quantitative approaches, i.e. for estimating the volume fraction of wastewater within a water body. Therefore, the stability of ACE at a specific site needs to be confirmed by the additional analysis of other proposed wastewater tracers such as carbamazepine, primidone, sucralose, gabapentin, oxypurinol, valsartan acid, and inorganic ions like chloride (Funke et al., 2015, Jekel et al., 2015, Nödler et al., 2013). Evaluating concentration ratios of such a broad set of potential wastewater tracers would help to gather information about the stability of the individual substances and thus allows for choosing the most reliable tracer compound in a particular environment.

The first results of the current study indicate that SA might be an excellent alternative to ACE as a conservative wastewater tracer due to its high polarity and negative charge, its elevated concentrations in WWTP effluents in the mg/L range and its biological and chemical stability observed in contact with activated sludge as well as filter sand from a water works. However, follow-up studies are needed to further confirm i) the ubiquitous presence in WWTP effluents as well as wastewater-impacted rivers and groundwater, ii) the conservative behavior, iii) the source specificity, i.e. that the major portion of SA concentrations originates from wastewater and iv) that transformation of ACE to SA within the respective environmental compartment is quantitatively negligible.

## 4 Conclusions

• The degradation of ACE in municipal WWTPs is not as rare as previously expected. A pronounced ACE degradation can also be observed in slow sandfilters fed with wastewater-influenced surface water.
• Neither was ACE removal enhanced in reactors with increased nitrification rate, nor did the initial ammonium concentration or the inhibition of AMO affect the degradation rate.
• Degradation of ACE in activated sludge can occur under oxic as well as denitrifying conditions, whereas no ACE degradation was observed under anaerobic conditions in the absence of both dissolved oxygen and nitrate.
• The main biotransformation product of ACE is SA. Further organic TPs are only formed in traces and a rapid removal of the organic part of the ACE molecule occurs.
• First results indicate no removal of SA by biological treatment in WWTPs. The main fraction of SA discharged from municipal WWTPs can be expected to originate from its extensive use in acid cleaning agents rather than the degradation of ACE.
• In the last decade, ACE has been proposed as a conservative wastewater tracer. However, the results of this study revealed that ACE is not generally persistent and thus its stability in the respective environment needs to be confirmed by the simultaneous quantification of additional tracer compounds. The absence of ACE in a water body is not necessarily associated with the absence of domestic wastewater.
• SA might be a promising wastewater tracer, due to its elevated polarity and microbial and chemical stability.

## Figures and Tables

**Table 1:** Overview of the different test setups in laboratory batch experiments.

Setup	Inoculum	Matrix	Treatment^a^	ACE conc.
A1	Activated sludge (WWTP)	Influent WWTP	Aerobic	50 μg/L
A2	Activated sludge (WWTP)	Influent WWTP	Aerobic, ATU-addition (5 mg/L)	50 μg/L
A3	Activated sludge (WWTP)	Effluent WWTP	Aerobic	50 μg/L
A4	Activated sludge (WWTP)	Effluent WWTP	Aerobic, NH_4_-N-addition (20 mg/L)	50 μg/L
A5	Activated sludge (WWTP)	Influent WWTP	Aerobic, autoclaved	50 μg/L

B1	Activated sludge (WWTP)	Effluent WWTP	Aerobic	30 μg/L
B2	Activated sludge (WWTP)	Effluent WWTP	Anoxic, NO_3_-N-addition (200 mg/L)	30 μg/L
B3	Activated sludge (WWTP)	Effluent WWTP	Anaerobic, no aeration	30 μg/L

C1	Activated sludge (R1)	Effluent WWTP	Aerobic	40 mg/L
C2	Activated sludge (R1)	Effluent WWTP	Aerobic	No spike

D1	Activated sludge (R1)	Phosphate buffer	Aerobic	400 μg/L
D2	Activated sludge (R1)	Phosphate buffer	Aerobic	b

E1	Filter sand	Deionized water + potable water (80/20, v/v)	Aerobic	10 μg/L
E2	Filter sand		Aerobic	10 mg/L

a Aeration in aerobic experiments was checked at least twice a day and air flow was controlled with a rotameter. In setups with activated sludge complete mixing was ensured and intended redox conditions were confirmed by measurement of NO_3_-N (Data provided in the SI) as indicator, since NO_3_^−^ is formed via nitrification under aerobic conditions, depleted via denitrification under anoxic conditions and absent under anaerobic conditions.

b Spiked with 400 μg/L ANSA.
