# Supplementary material for: Biodegradation of the artificial sweetener acesulfame in biological wastewater treatment and sandfilters
Source: Water Res. 2017 Mar 1;110:342–53. doi: 10.1016/j.watres.2016.11.041 (PMC5292994; doi:10.1016/j.watres.2016.11.041)
Supplement: Supplementary file 1 [file mmc1.pdf]

Biodegradation of the artificial sweetener acesulfame in biological wastewater treatment and sandfilters

Sandro Castronovo[a,1], Arne Wick[a,1], Marco Scheurer[b], Karsten Nödler[b], Manoj Schulz[a], Thomas A. Ternes[a*]

[a] Federal Institute of Hydrology, Am Mainzer Tor 1, 56068 Koblenz, Germany

[b] DVGW Water Technology Center Karlsruhe (TZW), Department of Analyses and Water Quality, Karlsruher Str. 84, D-76139 Karlsruhe, Germany

[*] Corresponding author.
[1] These authors contributed equally to this work.

Supplementary Information

Outline	Chapter/Table/ Figure	Page
**Material and Methods**		
Information about the WWTPs	Tab. S1	S3
Quantitative analysis of ACE by LC-MS/MS	S1.1	S4
Quantitative analysis of ACE and the TPs SA and ANSA by IC-MS/MS	S1.2	S4-S5
Monitored mass transitions and compound specific MS parameters used for quantification of acesulfame	Tab. S2	S5
Calibration and determination of the limit of quantification	S1.3	S5
TP identification by high-resolution mass spectrometry	S1.4	S5-S6
Determination of basic operational parameters	S1.5	S6
**Results and discussion**		
Basic operational parameters measured in laboratory batch experiments for the approaches A and B	Tab. S3	S7
Basic operational parameters measured in laboratory batch experiments for the approach C	Tab. S4	S8
Basic operational parameters measured in bench scale reactors in batch mode	Tab. S5	S9
Basic operational parameters measured in influent and effluent of the bench scale reactors in sequencing batch mode,	Tab. S6	S10

Concentration [µg/L] of ACE in surface water and in the respective collection well after slow sand filtration and short underground passage.	Fig. S1	S11
Ratio [%] of the sum of molar concentrations of ACE and SA and the spiked ACE concentration (ACE+SA/spiked ACE) in the high spike batch experiment (c_0 (ACE) = 40 mg/L) with activated sludge.	Fig. S2	S11
Formation of SA by transformation of ANSA (c0 =400 µg/L) in batch experiments (n=3) conducted with activated sludge from SBR which was repeatedly washed and resuspended in 50 mM phosphate buffer.	Fig. S3	S12
Formation of SA by transformation of ACE (c0 =400 µg/L) in batch experiments (n=3) conducted with activated sludge from SBR which was repeatedly washed and resuspended in 50 mM phosphate buffer.	Fig. S4	S12
List of retention times (RTs) and precursors and product ions from MS and MS^2 spectra obtained from ACE and ACE TPs using IC-LTQ-Orbitrap-MS with electrospray ionization in negative ionization mode.	Tab. S7	S13
Identification of TPs	S2	S13-S14
Possible chemical structures of TP180a and TP 180b.	Fig. S5	S15
References	S3	S15

Tab. S1: Overview about the biological treatment steps, selected operational parameters as well as the average ACE concentrations (± standard deviation) detected in the influents and effluents of 13 municipal WWTPs which were sampled over a period of two weeks (24 h composite samples, n=6). The operational parameters were provided by the WWTPs. Values of the chemical oxygen demand (COD) and NH4-N represent either the annual average or (in parentheses) the average of the sampling period. Influent samples were taken from raw wastewater before biological treatment and effluent samples were taken from the final effluent before discharge into the receiving water (i.e. after secondary clarification or sand filtration).

WWTP	Capacity [PE]	Discharge [m^3/d]	Biological treatment steps	SRT [d]	TSS [g/L]	COD Influent [mg/L]	COD Effluent [mg/L]	COD Removal [%]	NH4-N Influent [mg/L]	NH4-N Effluent [mg/L]	NH4-N Removal [%]	ACE Influent [µg/L]	ACE Effluent [µg/L]	ACE Removal [%]
A	50,000	13,000	AS (pD, N)	11	ND	376	18	95.2	24	1.0	95.8	32 ± 9	1.0 ± 0.1	96 ± 3
B	44,500	12,000	AS (pD, N), SF	20	ND	(650)	(13)	(98)	(37)	(<0.1)	(>99.7)	46 ± 6	1.4 ± 0.3	97 ± 1
C	50,000	8100	AS (pD, N)	20	3.5	319	20	93.7	28	0.23	99.2	28 ± 6	0.97 ± 0.21	96 ± 1
D	47,500	7500	AS (pD, N)	12	4	639	24	96.2	34	0.54	98.4	35 ± 7	2.2 ± 0.2	93 ± 2
E	30,000	6130	AS-s (iD, N)	29	6	437	18	95.9	27	0.2	99.3	39 ± 10	2.7 ± 0.3	92 ± 4
F	80,000	62,400	AS (pD, N)	17	3.8	496 (321)	18 (16)	96.4 (95.0)	26	0.7	96.3	26 ± 7	1.9 ± 0.2	92 ± 3
G	78,000	8500	AS (pD, N), SF	11	ND	879	26	97.0	19	0.51	97.3	20 ± 5	1.7 ± 0.7	90 ± 6
H	54,000	25,000	AS (pD, N), FB (pD, N), SF	10	ND	650	23	96.5	20	0.29	98.6	25 ± 9	2.8 ± 1.1	86 ± 11
I	29,000	4420	AS (pD, N)	ND	3.5	(370)	(22)	(94.1)	(54)	(<0.1)	(>99.8)	36 ± 5	4.8 ± 0.5	87 ± 1
J	440,000	88,400	AS (pD, N), SF	14	4.5	(527)	(15)	(97.4)	ND	ND	ND	26 ± 3	3.1 ± 0.2	88 ± 1
K	350,000	60,300	AS (iD, N)	12	3.5	440 (359)	33 (31)	92.5 (91.2)	50	4.9	90.3	81 ± 28	18 ± 14	77 ± 13
L	220,000	27,400	AS (pD, N)	13.5	4	703	37	94.7	40	0.56	98.6	48 ± 2	13 ± 1	72 ± 2
M	25,500	3200	H-BF-AS (pD, N)	5	ND	(121)	(19)	(84.2)	(34)	(0.13)	(99.6)	43 ± 6	19 ± 3	57 ± 8

AS: activated sludge treatment, AS-s: activated sludge treatment with sludge stabilization, pD: pre-denitrification, iD: intermittent denitrification, N: nitrification, FB: fixed-bed biofilm reactor, H-BF-AS hybrid biofilm-activated sludge process, SF: sand filtration; ND: no data.

S1 Description of the analytical methods

S1.1 Quantitative analysis of ACE by LC-MS/MS

Quantitative analysis of ACE in samples from WWTPs and the waterworks was conducted by LC-MS/MS. After addition of the surrogate standard (4 µg/L ACE-d_4), 80 µL of the samples were injected into an Agilent 1260 Series liquid chromatography system (Agilent Technologies, Waldbronn, Germany) coupled to a SCIEX QTrap 5500 mass spectrometer (SCIEX, Darmstadt, Germany). Chromatographic separation was achieved using a Zorbax Eclipse Plus C18 column (2.1 x 150 mm, 3.5 µm, Agilent Technologies, Waldbronn, Germany). Ultrapure water was used as mobile phase A and methanol as mobile phase B, both acidified with 0.1% formic acid. The gradient elution applied was as follows: 100% A for 1 min, decrease to 80% A within 1 min, further decrease to 0% A within 14.5 min, hold isocratic at 0% A for 5.5 min, increase to initial conditions (100% A) within 0.1 min and hold isocratic for 6 min. The total run time was 28 min and the flow rate and the column temperature were set to 0.3 mL/min and to 30°C, respectively. The mass spectrometer was operated with negative ion electrospray ionization (ESI) using multiple reaction monitoring (MRM). The ion source conditions were as follows: collision gas, medium; curtain gas 40 psi; ion source gas 1, 40 psi; ion source gas 2, 45 psi; source temperature 500°C; ion spray voltage, -4.5 kV; entrance potential, -10 V. The monitored mass transitions and compound specific MS parameters are described in Tab. S2.

S1.2 Quantitative analysis of ACE and the TPs SA and ANSA by IC-MS/MS

Quantitative analysis of ACE and the TPs SA and ANSA in samples from batch experiments was realized with an ion chromatography system (Metrohm 881 Compact IC pro with chemical suppressor, Metrohm GmbH, Filderstadt, Germany), coupled to an API 4000 triple quadrupole mass spectrometer (SCIEX, Darmstadt, Germany). ACE-d_4 was used as surrogate standard. Chromatographic separation was achieved on a Metrosep A Supp 5 anion exchange column (150 x 4 mm) connected to a Metrosep A Supp 4/5 guard column (Metrohm GmbH, Filderstadt, Germany). Ultrapure water and a carbonate buffer (5 mM Na_2CO_3 and 3.1 mM $NaHCO_3$) both amended with 10% methanol were used as mobile phase A and B, respectively. The gradient elution applied was as follows 36% A for 8 min, decrease to 0% A within 2 min, hold isocratic at 0% A for 30 min, increase to initial conditions (36% A) within 1 min and hold isocratic at 36% A for 4 min. The total run time was 45 min and the flow rate and the column temperature were set to 0.7 mL/min and to 50°C, respectively. The injection volume was 10 µL. The mass spectrometer was operated with negative electrospray ionization (ESI) using multiple reaction monitoring (MRM). The ion source conditions were as follows: collision gas, medium; curtain

gas 30 psi; ion source gas 1, 50 psi; ion source gas 2, 50 psi; source temperature 600°C; ion spray voltage, -4.2 kV; entrance potential, -10 V. The monitored mass transitions and compound specific MS parameters are described in Tab. S2.

Tab. S2: Monitored mass transitions and compound specific MS parameters used for quantification of acesulfame (ACE), sulfamic acid (SA) and acetoacetamide-N-sulfonic acid (ANSA).

Compound	MRM 1 [m/z]	MRM 2 [m/z]	Dwell time [ms]	DP [V]	CE [eV]	CXP [V]
ACE	162.0/82.0	162.0/78.0	30	-50/-50	-22/-38	-5/-3
ACE-d4	166.0/86.0	-	30	-50/-	-22/-	-5/-
SA	96.0/80.0	-	30	-50/-	-24/-	-6/-
ANSA	180.0/96.0	180.0/80.0	30	-40/-40	-23/-55	-5/-10

DP: declustering potential, CE: collision energy, CXP: cell exit potential

S1.3 Calibration and determination of the limit of quantification (LOQ)

For quantification of ACE, SA and ANSA an internal standard calibration with linear fitting and a weighing factor of 1/x was used. The limit of quantification was derived from the signal-to-noise (S/N) ratio in the native samples. At the LOQ, the S/N ratio of the mass transitions used for quantification (MRM 1) and confirmation (MRM 2, not available for SA) had to be at least 10 and 3, respectively.

S1.4 TP identification by high-resolution mass spectrometry

High-resolution mass spectra and MS^2 fragmentation experiments for the detection, identification and characterization of ACE TPs were obtained by Orbitrap-MS (LTQ Orbitrap Elite, Thermo Scientific, Bremen, Germany) operated in negative and positive ionization mode. The Orbitrap system was coupled to an ion chromatography system (Metrohm 881 Compact IC pro with chemical suppressor, Metrohm GmbH, Filderstadt, Germany). Chromatographic conditions were the same as described above (S.1.2) for quantification of the TPs SA and ANSA.

The source parameters for the IC-LTQ-Orbitrap-MS measurements were set as follows (values for the positive ionization mode are given in parenthesis): capillary temperature, 325°C (325°C); capillary voltage, 3.2 kV (2.8 kV); heater temperature, 450°C (350°C); sheath gas flow rate, 45 AU (40 AU); aux gas flow rate, 15 AU (15 AU); S-lens RF level, 60% (60%).

Data dependent acquisition was used to give rise to spectra as follows: A full scan (50–300 m/z) was performed followed by MS^2 scans for the two most intense ions with intensities of > 1000 and > 500, respectively. CID (collision-induced dissociation) and HCD (higher energy collisional dissociation) with normalized collision energies of 35% and 120%, respectively,

were used for fragmentation. In addition, dynamic exclusion was applied (exclusion of masses for which two MS^2 experiments have been performed; exclusion duration, 15 s; repeat duration, 30 s). External calibration was performed before the analysis of each batch to assure accurate mass determinations with a resolution of 60,000.

To avoid the missing of TPs not amenable to chromatographic separation by IC, the Orbitrap system was also interfaced with a Thermo Scientific Accela U-HPLC system (Accela pump and autosampler) equipped with a reversed-phase (RP) column (Hydro-RP, 150 x 4.6 mm, 4 µm) from Phenomenex (Aschaffenburg, Germany). Ultrapure water and acetonitrile (both amended with 0.1% formic acid) were used as mobile phase A and B, respectively. The gradient elution applied was as follows 100% A for 5 min, decrease to 60% A within 10 min, further decrease to 5% A within 3 min, hold isocratic at 5% A for 5 min, increase to initial conditions (100% A) within 0.1 min and hold isocratic at 100% A for 6.9 min. The total run time was 30 min and the flow rate and the column temperature were set to 0.5 mL/min and to 50°C, respectively. The injection volume was 10 µL.

S1.5 Determination of basic operational parameters

Basic operational parameters in experiments with activated sludge were measured with a spectral photometer (DR 5000 UV-VIS, Hach Lange GmbH, Düsseldorf, Germany) using cuvette tests (NO_3-N: LCK 339, NH_4-N: LCK303 and LCK304, DOC: LCK385, all Hach Lange GmbH, Düsseldorf, Germany).

In batch-experiments with sand, analyses of dissolved organic carbon (DOC) was performed according to German standard DIN EN 1484 (DIN, 1997) by combustion catalytic oxidation using a TOC analyser (TOC-L series, Shimadzu, Kyoto, Japan).

Tab. S3: Basic operational parameters measured in laboratory batch experiments for the approaches A and B (all concentrations as mg/L).

Setup	time	replicate									
		1			2			3			
	[h]	NH_4-N	NO_3-N	DOC	NH_4-N	NO_3-N	DOC	NH_4-N	NO_3-N	DOC	TSS
A1	0	15.3	1.4	14.7	15.3	1.5	15.4	-	-	-	1900
	4	< LOQ	13.8	-	1.7	9.3	-	-	-	-	
	6	< LOQ	15.6	11.8	< LOQ	13.9	-	-	-	-	
	27	< LOQ	27.4	-	< LOQ	25.9	-	-	-	-	
A2	0	15.0	1.2	17.3	14.8	1.2	16.2	-	-	-	
	4	-	-	-	-	-	-	-	-	-	
	6	15.8	1.0	14.1	15.8	-	-	15.7	-	-	
	27	20.2	2.9	-	17.3	2.8	-	-	-	-	
A3	0	< LOQ	8.0	11.9	< LOQ	7.8	12.5	-	-	-	
	4	-	-	-	-	-	-	-	-	-	
	6	< LOQ	9.9	13.4	< LOQ	9.9	-	-	-	-	
	27	< LOQ	23.9	-	< LOQ	25.4	-	-	-	-	
A4	0	19.3	7.6	12.6	19.5	7.6	11.7	-	-	-	
	4	< LOQ	24.2	-	4.1	18.7	-	-	-	-	
	6	< LOQ	26.2	10.9	< LOQ	23.6	-	-	-	-	
	27	< LOQ	39.8	-	< LOQ	35.6	-	-	-	-	
A5	0	3.1	158.0	16.8	3.3	155.0	17.0	-	-	-	
	4	3.0	161.0	16.6	3.3	151.0	17.0	-	-	-	
	6	-	-	-	-	-	-	-	-	-	
	27	3.1	-	-	3.2	-	-	-	-	-	
B1	0	-	< LOQ	-	-	< LOQ	-	-	< LOQ	-	1640
	8	-	-	-	-	-	-	-	-	-	
	24	-	-	-	-	-	-	-	-	-	
	48	-	26.3	-	-	27.7	-	-	25.9	-	
	96	-	37.8	-	-	42.3	-	-	37.0	-	
B2	0	-	204	-	-	211	-	-	214	-	
	8	-	188	-	-	192	-	-	197	-	
	24	-	176	-	-	172	-	-	176	-	
	48	-	150	-	-	147	-	-	153	-	
	96	-	117	-	-	116	-	-	118	-	
B3	0	-	< LOQ	-	-	< LOQ	-	-	< LOQ	-	
	8	-	< LOQ	-	-	< LOQ	-	-	< LOQ	-	
	24	-	-	-	-	-	-	-	-	-	
	48	-	< LOQ	-	-	< LOQ	-	-	< LOQ	-	
	96	-	< LOQ	-	-	< LOQ	-	-	< LOQ	-	

Tab. S4: Basic operational parameters measured in laboratory batch experiments for the approach C (all concentrations as mg/L).

Setup	time		replicate								
			1			2			3		
	[d]	[h]	NH_4-N	NO_3-N	DOC	NH_4-N	NO_3-N	DOC	NH_4-N	NO_3-N	DOC
Matrix C1 & C2 *	0	0				0.1	4.96	12.9			
C1	0	2	-	-	13.3	-	-	12.4	-	-	12.8
	2	48	< LOQ	24.7	12.9	< LOQ	25.28	13.2	< LOQ	24.7	14.1
	6	144	< LOQ	52.6	14.4	0.1	56	17.4	0.1	55.9	16.5
	10	240	-	-	18.4	-	-	21.8	-	-	20.8
	10	241	-	-	18.4	-	-	21.8	-	-	20.8
	14	336	1.7	76.6	17.1	4.1	77.7	20.8	2.4	77.4	19.7
	14	337	-	-	17.1	-	-	20.8	-	-	19.7
	16	384	-	-	18.8	-	-	19.7	-	-	20.4
	20	481	-	-	19.2	-	-	19.8	-	-	17.6
	21**	505	0.2	94.6	19.8	0.2	86.1	19.7	0.4	90.4	21.8
	23	552	-	-	19.8	-	-	17.1	-	-	19.8
	28	672	0.1	106	16.3	0.1	95.3	17.6	0.1	98.9	17.1
	29	696	-	-	22.1	-	-	23.2	-	-	27.2
C2	0	2	-	-	25.4	-	-	24.9	-	-	25.1
	2	48	< LOQ	27.0	25.6	< LOQ	25.8	25.7	< LOQ	26.1	25.5
	6	144	< LOQ	53.4	25.8	0.2	59.3	33.0	0.3	61	32.4
	10	240	-	-	24.2	-	-	40.4	-	-	41.0
	10	241	-	-	-	-	-	-	-	-	-
	14	336	0.4	75.2	17.4	6.2	78.4	35.8	5.4	-	35.4
	14	337	-	-	37.6	-	-	44.4	-	-	47.6
	16	384	-	-	34.9	-	-	42.4	-	-	40.8
	20	481	-	-	21.8	-	-	28.4	-	-	25.2
	21**	505	< LOQ	86.9	39.8	0.1	91.7	45.2	1.3	-	40.8
	23	552	-	-	43.6	-	-	39.8	-	-	41.2
	28	672	< LOQ	96.1	18.2	0.6	99.4	21.7	0.0	-	26.6
	29	696	-	-	23.7	-	-	26.3	-	-	29.8

*before aliquoting into separate test-vessels
** after respike

Tab. S5: Basic operational parameters measured in bench scale reactors in batch mode (all concentrations as mg/L).

Setup	time [h]	NH_4-N	NO_3-N	DOC
R1	0	11.8	1.7	19.5
	1	< LOQ	11.9	13.6
	2	< LOQ	12.3	12.9
	4	< LOQ	12.7	13.3
	7	< LOQ	14.0	13.1
	11	< LOQ	16.1	13.3
	24	< LOQ	24.6	13.9
	51	< LOQ	44	14.9
R2	0	12.0	0.3	18.1
	1	< LOQ	12.5	12.8
	2	< LOQ	12.8	12.5
	4	-	-	-
	7	-	18.7	-
	11	-	21.6	11.9
	24	< LOQ	33.3	13.1
	51	< LOQ	62.1	15.5
R3	0	7.8	210	12.9
	1	-	222	12.5
	2	-	235	12.6
	4	-	264	12.4
	7	6.7	298	12.6
	11	-	348	11.1
	24	-	504	11.3
	51	13.2	806	11.9
R4	0	< LOQ	10.8	11.9
	1	< LOQ	11.6	10.3
	2	< LOQ	11.8	10.4
	4	-	-	-
	7	-	15.3	-
	11	-	15.4	9.7
	24	< LOQ	15.9	9.5
	51	< LOQ	20.6	8.7

Tab. S6: Basic operational parameters measured in influent and effluent of the bench scale reactors in sequencing batch mode (all concentrations as mg/L).

Date	Influent		R1 effluent			R2 effluent			R3 effluent			R4 effluent		
	NH_4-N	DOC	NH_4-N	NO_3-N	DOC	NH_4-N	NO_3-N	DOC	NH_4-N	NO_3-N	DOC	NH_4-N	NO_3-N	DOC
23.03.2015	43.8	89.6	< LOQ	10.5	10.7	0.32	9.6	11.8	5.4	216	10.1	< LOQ	17.9	10.6
26.03.2015	49.0	85.2	< LOQ	12.0	11.2	0.47	9.7	12.2	4.9	230	10.0	< LOQ	15.0	10.3
09.04.2015	47.4	91.2	< LOQ	11.9	11.1	0.21	8.8	11.4	6.7	243	10.6	< LOQ	11.4	9.4
13.04.2015	46.2	72.8	0.09	11.4	11.2	0.26	9.2	12.3	13.8	218	11.8	< LOQ	-	9.9
24.04.2015	48.0	87.2	0.23	11.4	12.4	< LOQ	10.9	12.0	17.9	221	10.8	< LOQ	12.7	10.6
27.04.2015	45.0	87.6	0.30	10.6	11.7	< LOQ	10.0	12.1	12.6	231	12.4	< LOQ	12.9	11.2
25.05.2015	48.0	-	0.28	10.3	-	0.10	9.2	-	16.9	260	-	< LOQ	11.2	-
01.06.2015	36.6	70.2	0.17	7.3	10.1	0.19	6.5	11.8	17.1	245	10.4	< LOQ	9.3	9.8
05.06.2015	-	-	-	8.2	-	-	7.0	-	-	250	-	-	9.3	-
08.06.2015	59.8	73.4	0.24	-	11.5	0.22	-	12.4	17.8	-	10.8	< LOQ	-	11.0
13.07.2015	46.0	77.2	0.20	8.0	10.5	< LOQ	6.1	11.3	11.8	255	10.8	< LOQ	8.4	9.6
18.12.2015	35.8	71.6	< LOQ	8.4	11.1	< LOQ	7.6	12.7	20.4	188	10.3	< LOQ	6.7	9.4
21.12.2015	51.4	99.2	< LOQ	9.6	11.9	0.14	8.3	15.7	19.6	198	10.3	< LOQ	7.8	10.9

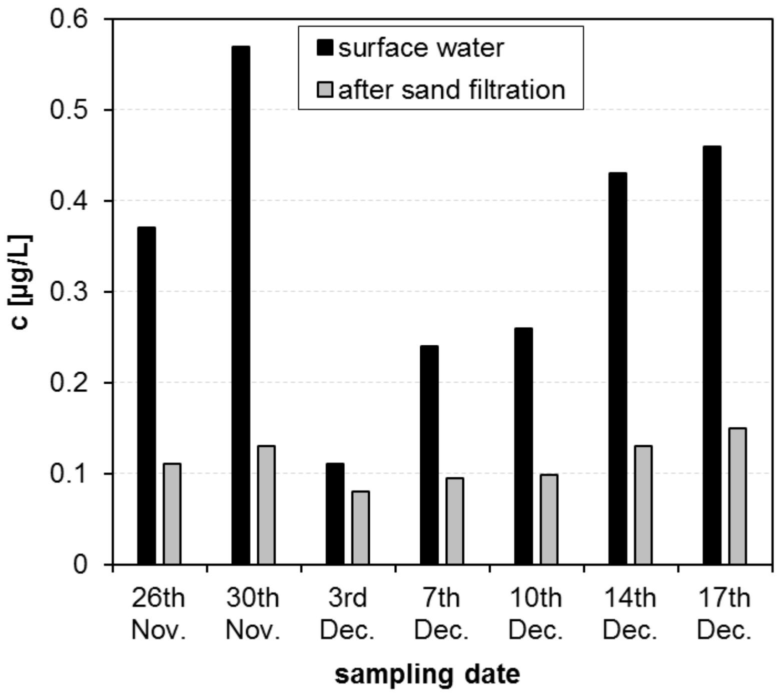

Fig. S1: Concentration [µg/L] of ACE in surface water and in the respective collection well after slow sand filtration and short underground passage.

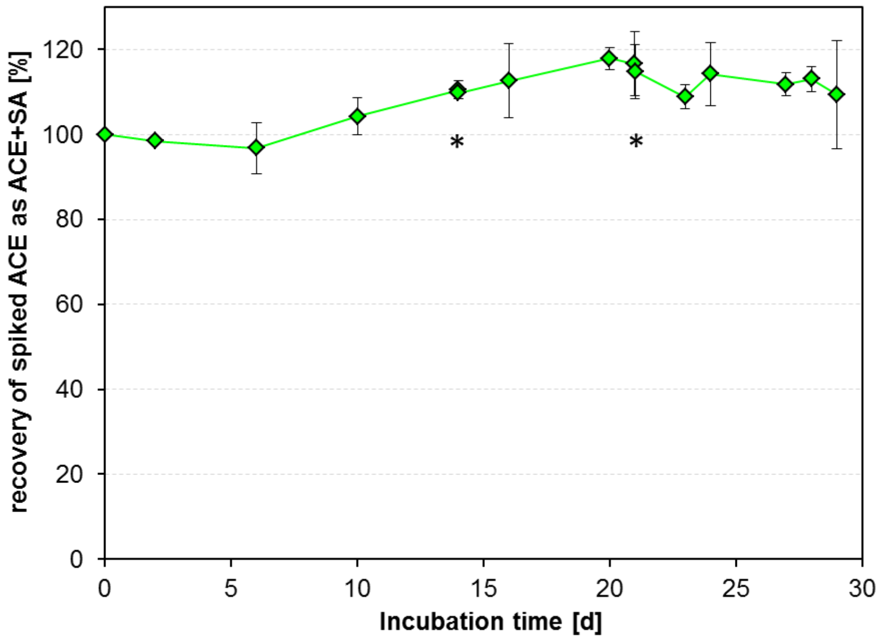

Fig. S2: Ratio [%] of the sum of molar concentrations of ACE and SA and the spiked ACE concentration (ACE+SA/spiked ACE) in the high spike batch experiment (c_0 (ACE) = 40 mg/L) with activated sludge. The error bars represent the standard deviation (n=3). *respike of ACE

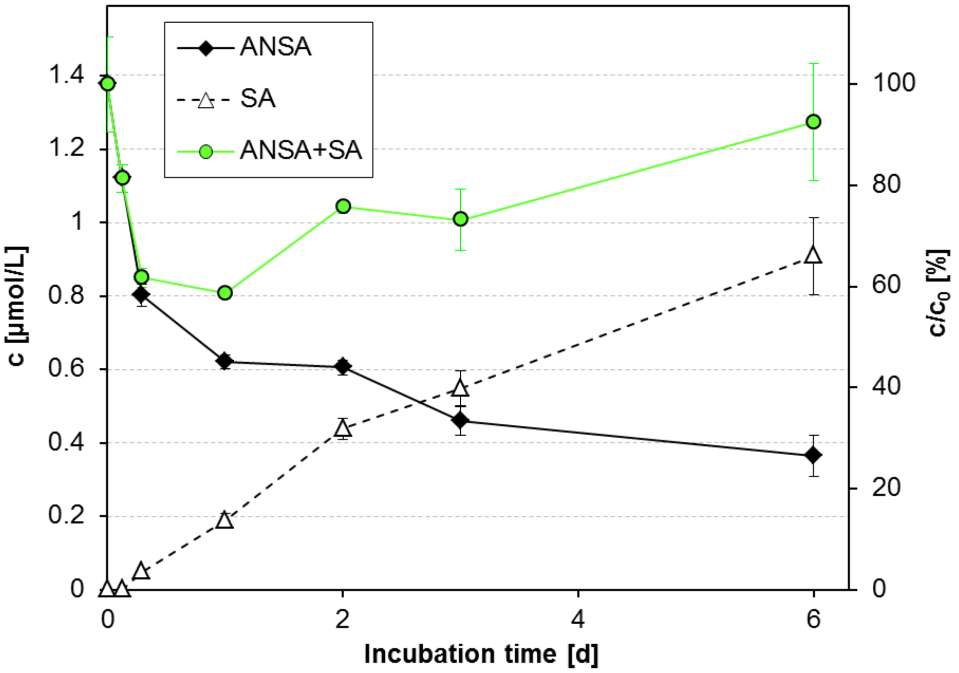

Fig. S3: Formation of SA by transformation of ANSA (c_0 =400 µg/L) in batch experiments (n=3) conducted with activated sludge from SBR which was repeatedly washed and resuspended in 50 mM phosphate buffer. The error bars represent the standard deviation.

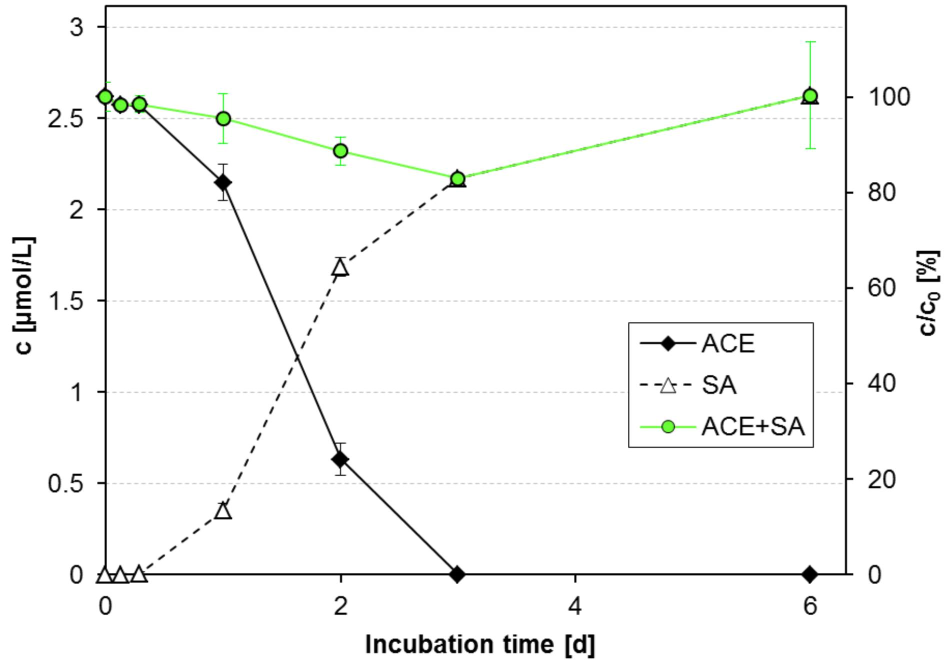

Fig. S4: Formation of SA by transformation of ACE (c_0 =400 µg/L) in batch experiments (n=3) conducted with activated sludge from SBR which was repeatedly washed and resuspended in 50 mM phosphate buffer. The error bars represent the standard deviation.

Tab. S7: List of retention times (RTs) and precursors and product ions from MS and MS^2 spectra obtained from ACE and ACE TPs using IC-LTQ-Orbitrap-MS with electrospray ionization in negative ionization mode.

**Compound**	**Precursor ion**	**Fragment ions**				**Proposed fragmentation**
Retention time (RT in min)	Mass/Charge (Error in ppm) Elemental comp.	Measured MW (Error in ppm)	Elemental composition	Intensity [%]	Fragmentation technique	
**Acesulfame**	161.9867 (0.546) $C_4H_4NO_4S$	101.9763 (1.5)	C_2NO_2S	3	CID (35%)	$[M-C_2H_4O_2]^-$
		98.0249 (1.8)	$C_4H_4NO_2$	3	CID (35%)	$[M-SO_2]^-$
		82.0300 (2.4)	C_4H_4NO	100	CID (35%)	$[M-SO_3]^-$
**TP 96** (5.9)	95.9763 (2.2) H_2NO_3S	79.9579 (6.5)		6	HCD (100%)	$[M-H-NH_2]^-$
**TP 180a** (23.8)	179.9972 (-0.04) $C_4H_6NO_5S$	121.9558 (2.6)	CNO_4S	2	CID (35%)	$[M-C_3H_6O]^-$
		95.9765 (3.8)	H_2NO_3S	100	CID (35%)	$[M-C_4H_4O_2]^-$
**TP 180b** (10.7)	179.9971 (-0.42) $C_4H_6NO_5S$	135.9711 (0.36)	$C_2H_2NO_4S$	100	CID (35%)	$[M-C_2H_4O]^-$
		121.9555 (1.0)	CNO_4S	25	CID (35%)	$[M-C_3H_6O]^-$
		105.9606 (1.2)	CNO_3S	35	CID (35%)	$[M-C_3H_6O_2]^-$
		72.0093 (2.7)	$C_2H_2NO_2$	35	CID (35%)	$[M-C_2H_4O_3S]^-$
**TP 178** (14.7)	177.9814 (-0.65) $C_4H_4NO_5S$	134.9758 (0.43)	$C_3H_3O_4S$	4	CID (35%)	$[M-CHNO]^-$
		98.0249 (1.4)	$C_4H_4NO_2$	100	CID (35%)	$[M-SO_3]^-$
**TP 192** (29.0)	191.9613 (2.2) $C_4H_2NO_6S$	112.0045 (4.4)	$C_4H_2NO_3$	100	CID (35%)	$[M-SO_3]^-$
		84.0096 (5.6)	$C_3H_2NO_2$	1	CID (35%)	$[M-SO_3-CO]^-$

CID: collision-induced dissociation, HCD: higher energy collisional dissociation

S2 Identification of TPs

Based on the measured exact mass of m/z 95.9763 suggesting a sum formula of H_2NSO_3 **TP96** was identified as sulfamic acid (SA). This was confirmed by comparison of retention times and MS-fragmentation-spectra with the acquired reference standard of SA.

The high resolution mass spectrometric analysis of **TP180a** (RT 23.8 min) and **TP180b** (RT 10.7 min) revealed an exact mass of 179.9972 which indicated the introduction of an oxygen (+O) and two hydrogen (+2H) atoms. Four plausible structures (see Fig. S5) were assumed for TP180a and TP180b: i) a hydrolysis of the amide bond leading to an open ring structure with a carboxylic group at the former C-4 position and a sulfamate moiety ((2Z)-3-(sulfamoyloxy)but-2-enoic acid, structure A), ii) a water addition in α-position (C-5) forming 5-hydroxy-6-methyl-4-oxo-1,2,3-oxathiazinan-3-ide 2,2-dioxide (structure B), iii) a water addition in β-position (C-6) of the keto group forming 6-hydroxy-6-methyl-4-oxo-1,2,3-oxathiazinan-3-ide 2,2-dioxide

(structure C) and hydrolysis of the hemiacetal of structure C leading to acetoacetamide-N-sulfonic acid (ANSA, structure D). The latter compound could be obtained as reference standard and based on the comparison of the RT and MS spectrum TP180a could be identified as ANSA.

The MS^2 spectrum of TP180b was completely different from that of TP180a and to the best of our knowledge has not been described so far. The MS^2 spectrum exhibited the following fragments: m/z 135.9711, $C_2H_2NO_4S$, $[M-C_2H_4O]^-$; m/z 121.9555, CNO_4S, $[M-C_3H_6O]^-$; m/z 105.9606, CNO_3S, $[M-C_3H_6O_2]^-$; m/z 72.0093, $C_2H_2NO_2$, $[M-C_2H_4O_3S]^-$. The latter fragment indicated that the additional oxygen is located in α-position (C-5) of the keto group of ACE. Since this only applies to structure B, TP180b was tentatively identified as 5-hydroxy-6-methyl-4-oxo-1,2,3-oxathiazinan-3-ide 2,2-dioxide.

For **TP178** (RT 14.7 min) an exact mass of 177.9814 was determined and indicated an hydroxylation either at the C-5 position or at the C-7 position. The former would lead to 6-methyl-4,5-dioxo-1,2,3-oxathiazinan-3-ide 2,2-dioxide which differs from the assumed structure of TP180b only by a keto group instead of an alcohol group at the C-5 position. However, since the MS^2 spectrum of TP 178 only exhibited one dominant fragment (m/z 98.0249, $C_4H_4NO_2$, $[M-SO_3]^-$) which was comparable to that of acesulfame but not to any of those of TP180b, it was assumed that TP 178 still bears the double bond between C-5 and C-6 and is more likely hydroxylated at the C-7 position. It was therefore tentatively identified as 6-(hydroxymethyl)-4-oxo-4H-1,2,3-oxathiazin-3-ide 2,2-dioxide.

The measured exact mass of **TP192** (m/z 191.9613, $C_4H_2NO_6S$) suggested the introduction of two additional oxygens and the cleavage of 2 hydrogens. TP192 was also detected doubly charged (m/z 95.4769) which indicated the presence of an acidic moiety. However, the MS^2 spectrum gave no indication of a carboxyl group (no cleavage of CO_2 or CH_2O_2). Instead the only plausible structure was that of 6-formyl-4,5-dioxo-1,2,3-oxathiazinan-3-ide 2,2-dioxide exhibiting a 1,3-dicarbonyl moiety. The deprotonation of TP 192 leading to the double charged species in the ESI source can be explained by the formation of enolate forms and their stabilization by delocalization of the charge over three atoms. Except for SA, TP 192 was the only TP for which no further degradation was observed.

However, the proposed chemical structures of the minor TPs TP180b, TP178 and TP192 could not be further confirmed, since reference standards were not available and the quantities were too low for structural elucidation by NMR.

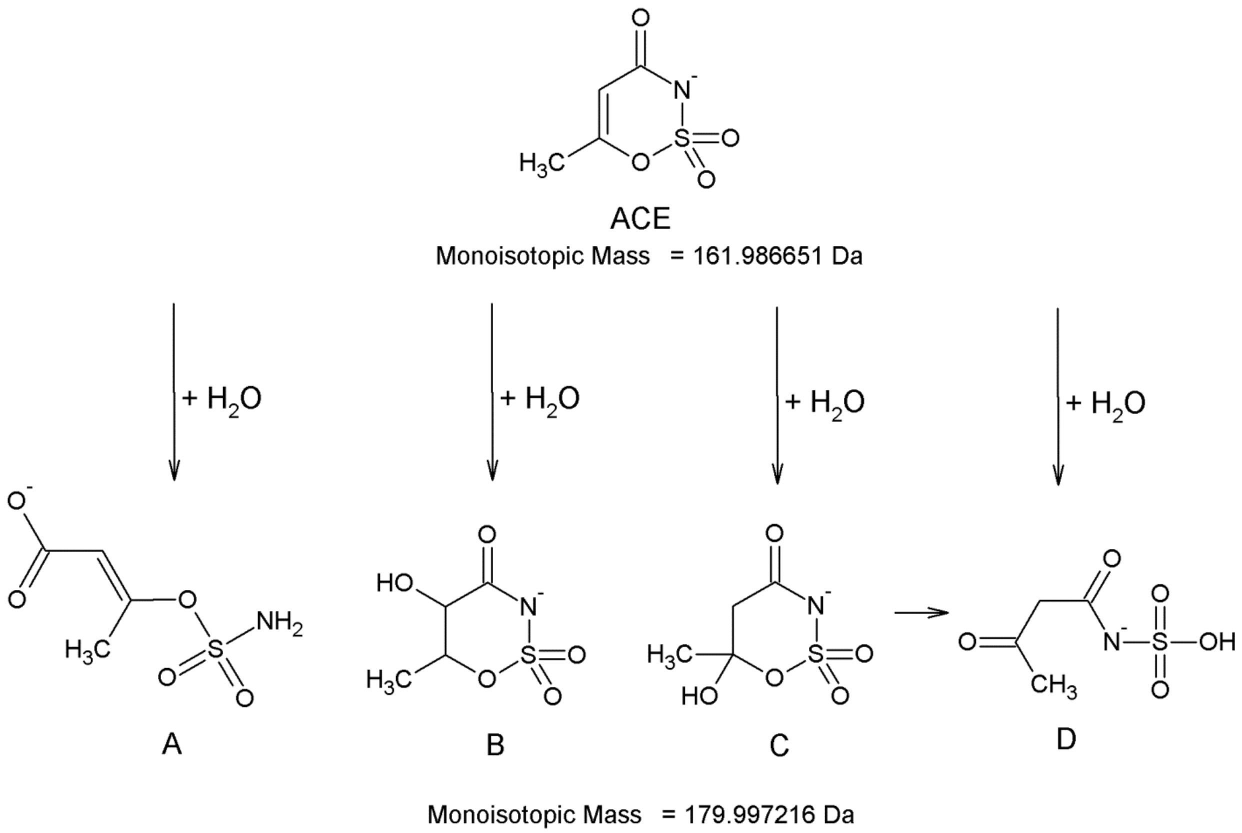

Fig. S5: Possible chemical structures of TP180a and TP 180b. TP 180a was unambiguously identified as structure D by comparison with a reference standard. TP 180b was tentatively identified as structure B.

S3 References

DIN, 1997. Water analysis - Guidelines for the determination of total organic carbon (TOC) and dissolved organic carbon (DOC); German version EN 1484-1997.
